# Depression in neurodegenerative disease: neurobiological mechanisms and emerging treatments

**DOI:** 10.1038/s41380-026-03728-8

**Published:** 2026-07-06

**Authors:** Harry Costello, Suzanne Reeves, Paul Glue, Allan H Young, Robert Howard

**Affiliations:** 1 Division of Psychiatry, University College London, London, UK; 2 Department of Psychological Medicine, University of Otago, Dunedin, New Zealand; 3 Division of Psychiatry, Department of Brain Sciences, Imperial College London, London, UK

**Keywords:** Depression, Neuroscience

## Abstract

Depression is one of the most common and disabling neuropsychiatric complications of neurodegenerative diseases. In Alzheimer’s disease (AD) and Parkinson’s disease (PD), depressive syndromes affect more than one-third of patients and are associated with accelerated cognitive decline, reduced quality of life, and increased healthcare utilisation. Despite this burden, current antidepressant treatments show little or no efficacy in these populations, suggesting that depression arising in the context of neurodegeneration may reflect distinct underlying neurobiological mechanisms. Emerging evidence from molecular imaging, neuropathology, and cognitive neuroscience indicates disease-specific disruption of monoaminergic, glutamatergic, inflammatory, and reward-related circuits in AD and PD. These alterations may undermine the mechanisms of action of standard antidepressants and contribute to treatment resistance. Yet patients with cognitive impairment are routinely excluded from antidepressant trials, and few high-quality studies have evaluated novel therapies in neurodegenerative disease. In this narrative review, we synthesise current evidence on the neurobiological mechanisms underpinning depression in AD and PD and critically evaluate emerging pharmacological and neuromodulatory therapies targeting glutamatergic, serotonergic, dopaminergic, immune, and circuit-level dysfunction. Here, we highlight that depression in neurodegenerative disease offers a unique and underutilised model for mechanistically guided antidepressant development. We identify how interventions including glutamate modulators, dopaminergic agents, kappa opioid antagonists, immune-modulating therapies, and next-generation brain stimulation approaches may offer therapeutic promise. By aligning treatment development with disease-specific circuit and molecular pathology, this framework may improve outcomes for this neglected population while advancing precision psychiatry more broadly.

## Introduction

Depression and neurodegenerative disease are two of the leading causes of disability worldwide and a major contributor to global disease burden, with rising prevalence. The intersection of these two conditions, depression in neurodegenerative disease, is a common, disabling, yet neglected clinical problem with profound effects on quality of life. In the two most common neurodegenerative conditions, Alzheimer’s disease (AD) and Parkinson’s disease (PD), depression affects 42 and 35% of patients respectively [1, 2]. Depression in these conditions predicts greater disease progression, poorer quality of life, higher mortality [3, 4], and doubles healthcare costs [5–8].

Beyond its symptomatic impact, depression may interact bidirectionally with neurodegenerative pathology and represents a potential target for prevention and disease modification [9]. Depression is both a risk factor for and prodromal feature of AD and PD [10, 11], doubling the risk of developing these conditions, and predicts more rapid cognitive decline (additional one point deterioration on the mini-mental state examination each year in AD) [12]. The 2024 Lancet Commission on dementia prevention estimated that eliminating depression could reduce dementia incidence by 4%, exceeding most other modifiable risk factors [13], and highlighting how earlier effective treatment may improve cognitive reserve, mitigating disease progression [9].

Despite this clinical importance, we lack effective pharmacological treatments for depression in neurodegenerative diseases [14]. Meta-analyses consistently demonstrate little evidence of clinical efficacy of existing antidepressants in AD or PD depression [15–18]. Yet around one-third of AD and PD patients are still prescribed an antidepressant [19, 20]. This disconnect between clinical burden and benefit highlights the clear clinical need for effective therapies.

**Fig. 1:**
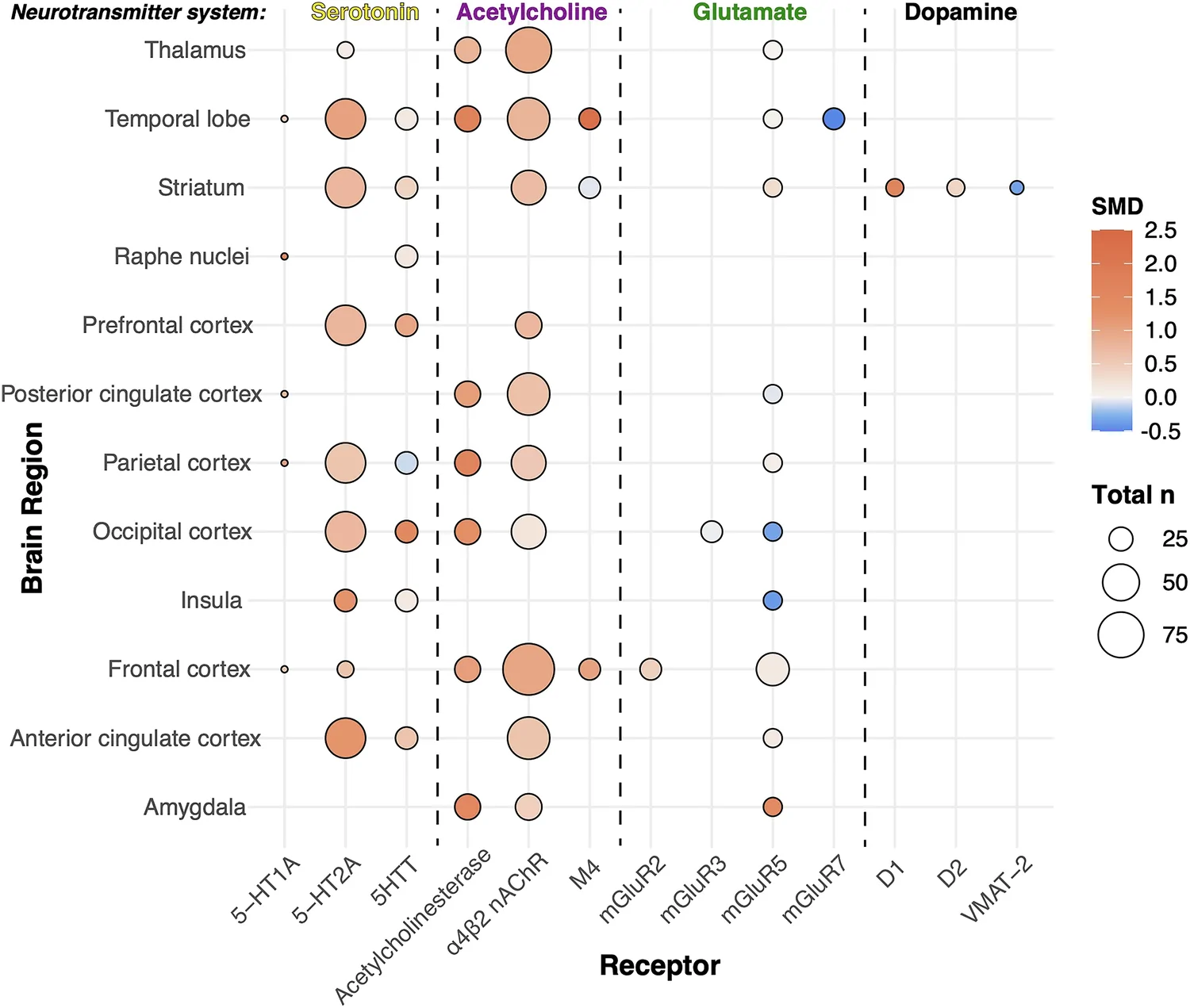
Pooled PET receptor and transporter binding differences in Alzheimer’s disease (AD) versus healthy controls (HC). Standardised mean differences (SMDs) reflect group differences in radioligand binding potential (BPND) between AD and HC. Positive SMD values indicate lower binding in AD relative to HC. Studies were identified through existing systematic reviews and meta-analyses of PET imaging in AD, with data verified against original publications (see Supplementary Methods and Table S1).

One explanation for this treatment failure is that depression in neurodegenerative disease may reflect disease-specific neurobiological mechanisms distinct from idiopathic depression, undermining the therapeutic mechanisms of current antidepressants [14]. Advances in molecular imaging, and systems neuroscience have demonstrated early and regionally specific disruption of monoaminergic, glutamatergic, inflammatory, and reward-related circuits in AD and PD. For example, as shown in Fig. 1., pooled analysis of existing positron emission tomography (PET) imaging studies in AD comparing receptor binding versus healthy controls reveal marked receptor- and region-specific alterations across neurotransmitter systems. While these findings do not establish causal mechanisms of treatment resistance, they are consistent with alterations in neurotransmitter system integrity that may influence antidepressant target engagement and action. In PD, depression is characterised by marked disruption in reward processing in comparison to late-life depression, worsening in the “off” dopaminergic medication state [21]. Such alterations may affect the neurobiological mechanisms upon which conventional antidepressants depend, and help explain reduced efficacy in late-life and prodromal depression [22].

At the same time, depression in neurodegenerative disease offers a uniquely informative model for understanding mood disorders. Unlike idiopathic depression, the temporal evolution of neurodegenerative diseases can be mapped against biomarker trajectories, neuroimaging changes, and cognitive decline. This allows depressive symptoms to be characterised across molecular, circuit and behavioural levels. Studying depression within this framework can therefore clarify mechanisms of treatment resistance and inform mechanistically guided antidepressant development.

**Fig. 2:**
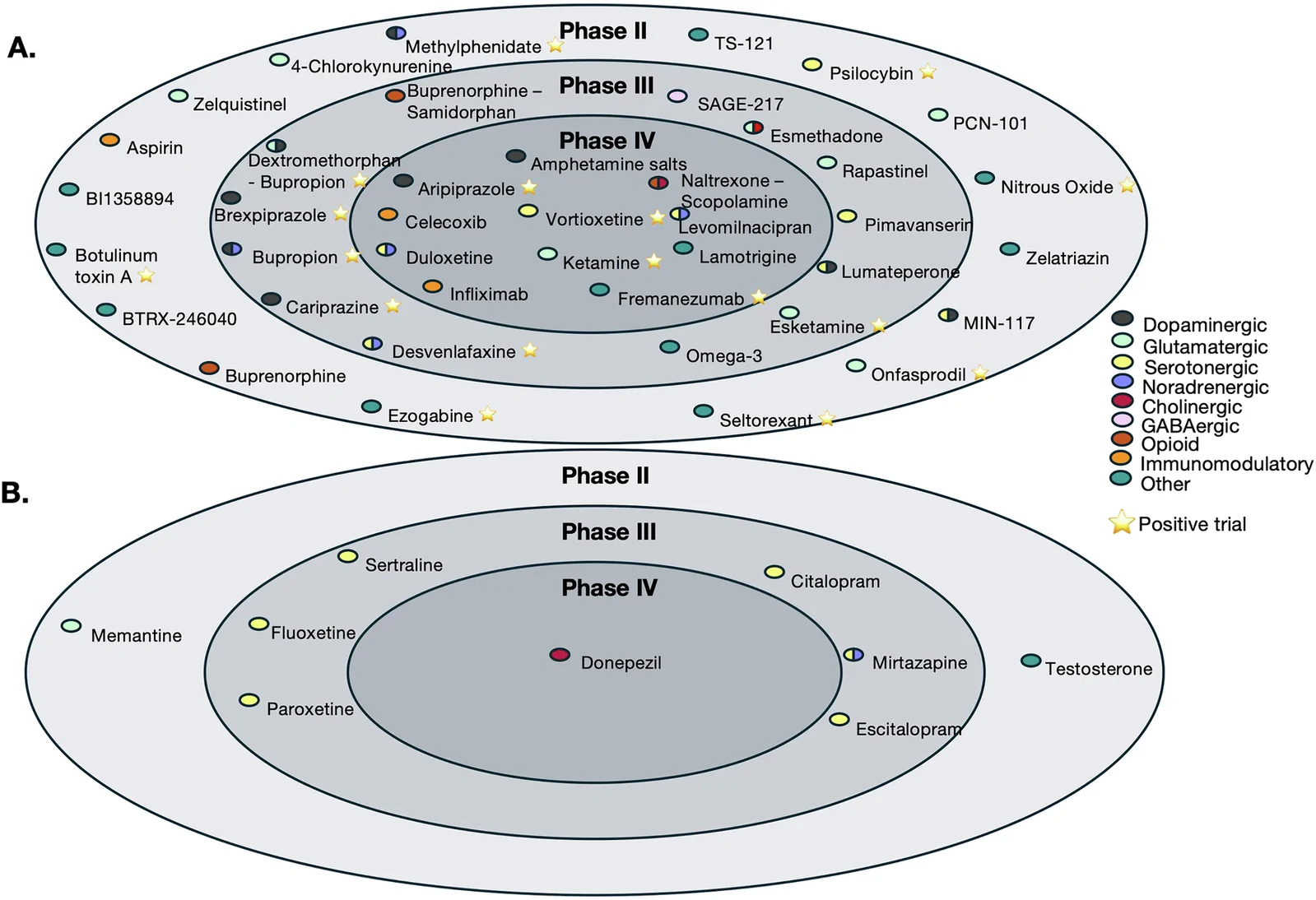
Clinical trial landscape for depression in older adults and patients with dementia. Phase II–IV interventional trials for depression registered on ClinicalTrials.gov (2014–present) including (**A**) older adults (≥65 years) and (**B**) patients with dementia. Concentric rings represent clinical trial phase. Drug classes are colour-coded by primary mechanistic target. A star indicates at least one reported positive primary outcome in the registered trial record or associated publication (see Supplementary Methods).

Despite growing mechanistic insight, a striking translational gap persists. Patients with neurodegenerative disease are frequently excluded from antidepressant trials, while depression is often overlooked in disease-modifying studies [23]. Consequently, few high-quality studies have evaluated emerging pharmacological or neuromodulatory therapies specifically for depression in AD or PD. This neglect persists despite the rapid therapeutic innovation in the broader field of depression, where multiple emerging therapies have demonstrated promising results in early-phase trials or have entered clinical practice. As shown in Fig. 2. a comparison of phase II–IV clinical trials for depression in older adults with trials registered for depression in dementia highlights the marked scarcity of studies and limited mechanistic diversity targeting this population (see Fig. 2). Evidence from trials conducted in younger adults cannot be assumed to generalise to older individuals with neurodegenerative diseases, where pharmacokinetic and pathophysiological factors differ substantially.

In this narrative review we examine the neurobiological mechanisms underpinning depression in AD and PD, and critically evaluate emerging pharmacological and neuromodulatory therapies. Targeted searches of MEDLINE, Embase, Web of Science, and ClinicalTrials.gov were conducted, focusing primarily on literature published since 2000, to integrate evidence across molecular, neurocognitive, and therapeutic domains. We prioritised human studies, particularly in vivo neuroimaging, translational neuroscience, and clinical trials, supplemented by key mechanistic work where relevant. Evidence was synthesised thematically across neurotransmitter systems, circuit-level dysfunction, and treatment mechanisms, with emphasis on convergent findings relevant to depression in neurodegenerative disease and disease-specific mechanisms.

By aligning treatment development with pathophysiology, we aim to reframe depression in neurodegenerative disease as a mechanistically distinct, disease-specific condition requiring tailored therapeutic approaches. Addressing this challenge has implications beyond AD and PD, and may improve outcomes in this neglected population while advancing more effective, precision psychiatry more broadly.

## Pharmacological therapies

### Glutamate modulating therapies

#### The role of the glutamatergic system in neurodegenerative disease and depression

Glutamate is the most abundant neurotransmitter in the brain and a key mediator of cognitive processes such as learning and memory which are disrupted in both depression and neurodegenerative disease [24].

Disrupted glutamatergic neurotransmission, and glutamate-induced neurotoxicity are hypothesised to be important pathological mechanisms in both AD and PD [25]. However, the nature and functional consequences of these alterations differ between disorders. In AD, post-mortem and in vivo evidence suggests region-specific glutamatergic dysregulation, including altered expression of the ionotropic glutamate receptor subtypes *N*-methyl-d-aspartate (NMDA) and α-amino-3-hydroxy-5-methyl-4-isoxazolepropionic acid (AMPA) receptors, and abnormal extracellular glutamate levels [25–29]. While late-stage post-mortem studies often demonstrate reduced receptor density consistent with neuronal loss, earlier in vivo studies indicate excessive NMDA receptor activation and increased glutamatergic tone [25–28]. Together, these findings support a model of early excitotoxic stress in AD followed by downstream synaptic and receptor loss [25–28].

In contrast, evidence for glutamatergic dysfunction in PD is more limited and largely preclinical, but indicates that increased glutamatergic activity within striatal circuits may contribute to both motor and non-motor symptoms, particularly in the context of chronic levodopa exposure [29]. Compensatory upregulation of glutamate transporters has been observed, and may underlie the clinical efficacy of amantadine, a dopaminergic agent with weak NMDA receptor antagonism, in the treatment of dyskinesia [30, 31].

In depression, glutamatergic abnormalities are also well documented. In vivo proton magnetic resonance spectroscopy (MRS) report reduced glutamate metabolites in the prefrontal cortex [32], while molecular imaging and post-mortem studies demonstrate elevated glutamate levels in the plasma, cerebrospinal fluid, and brain tissue of depressed patients [33, 34], Pre-clinical works suggests these alterations are more complex, regionally heterogeneous and interact with stress-related mechanisms which increase extracellular glutamate in medial PFC and hippocampus in the acute phase [35].

These differences may have implications for therapeutic targeting. In AD, glutamatergic dysfunction is linked to cortical network disruption and excitotoxicity, whereas in PD it interacts with dopaminergic treatment and deficits within frontostriatal circuits. Memantine, a partial NMDA receptor antagonist, produces modest cognitive benefits with marginal effects on mood symptoms in AD and in depression more broadly [36, 37]. However, newer glutamatergic agents have demonstrated clinically meaningful antidepressant effects, renewing interest in this pathway, although its relevance to AD and PD may differ according to underlying circuit pathology.

#### N-methyl-D-aspartate modulators

##### Ketamine

Over two decades have passed since the first placebo-controlled trial demonstrated the antidepressant effects of subanaesthetic ketamine [38]. Subsequent trials confirm that ketamine rapidly and robustly improves core depressive symptoms, particularly depressed mood, anhedonia and suicidal thoughts [39, 40]. Despite ketamine’s replicated antidepressant efficacy in treatment-resistant depression, it has not been evaluated in AD or PD, and there is currently no direct evidence for efficacy in neurodegenerative populations. Its relevance to these conditions is therefore inferential, based on its effects on glutamatergic signalling, synaptic plasticity, and reward processing, as well as downstream modulation of neurotrophic factors.

Pharmacologically ketamine, a non-competitive NMDAR antagonist, is composed of two enantiomers: (R)-ketamine and (S)-ketamine, collectively referred to as racemic ketamine. (S)-ketamine is approximately 3–4 times more potent at NMDA inhibition and is thought to mediate the acute dissociative effects, which led to the development of intranasal esketamine containing the isolated (S)-enantiomer [41]. However, emerging evidence suggests racemic ketamine may have greater antidepressant efficacy than esketamine, implicating a potential role for the longer-acting (R)-enantiomer and active metabolites in sustaining effects with fewer adverse events [41]. However, differences may also reflect higher dosing with racemic ketamine [41]. Ketamine undergoes extensive hepatic metabolism to norketamine and hydroxynorketamine (HNK), metabolites that persist beyond the parent compound’s short 2–4 h half-life and are largely excreted in urine [41].

A systematic review of 28 studies, including a meta-analysis of seven trials (*n* = 373), reported a large reduction in depressive symptoms at 24 h (SMD = −0.77, 95% CI−1.08 to −0.46), with smaller but sustained effects at 7 days (SMD = −0.49) [42]. Two further meta-analyses, each incorporating over 40 studies, found large sustained antidepressant effects of ketamine at 4 weeks with 2–3 weekly treatments (SMD = −0.85 [43] and −1.28 [44], respectively). However, most included trials had short follow-up periods, and the effect sizes may be inflated by small samples and open-label extensions [43, 44].

Clinical response rates in treatment-resistant depression (TRD) are also robust, with two meta-analyses finding large pooled odds ratios favouring ketamine over placebo (OR = 3.4, 95% CI = 2.14, 5.41; and OR = 6.3, 95% CI = 3.33, 12.05) [42, 45]. Only neuromodulatory interventions such as electroconvulsive therapy (ECT) and repetitive transcranial magnetic stimulation (rTMS) have demonstrated superior efficacy in comparative analyses [45].

Data in older adults are limited, and the only trial of esketamine nasal spray in this population did not demonstrate antidepressant efficacy [46]. This research gap in older adults may be due to concerns over the high rates of dissociation (~70%) induced by IV ketamine, as well as the risk of psychosis and perceptual disturbances. These risks are particularly relevant in PD and dementia, where baseline vulnerability to psychosis is elevated. Although typically dose-dependent and transient, these risks underscore the need for careful patient selection and monitoring in future trials.

Emerging evidence suggests that ketamine metabolites play a significant role in mediating its antidepressant effects [47, 48]. Oral ketamine extended-release formulations, which provide more sustained effects, have been shown to have comparable efficacy to intravenous infusions and may mitigate dissociation [49]. A phase II double-blind trial (*n* = 168) reported significant reductions in Montgomery–Åsberg Depression Rating Scale (MADRS) scores (LS mean difference −6.1, 95% CI 1.0–11.16) and a 50% lower relapse rate at 180 mg compared with placebo, with minimal dissociation (3–15%) or blood pressure changes [50]. Such formulations may offer a more practical and tolerable approach for AD and PD populations, though disease-specific trials are still lacking.

Mechanistically, ketamine is hypothesised to exert antidepressant effects through NMDA receptor blockade on GABAergic interneurons, enhancing AMPA-mediated glutamatergic throughput and activating downstream mTOR signalling pathways that promote synaptic plasticity [47]. Preclinical studies demonstrate that ketamine increases the synthesis of brain-derived neurotrophic factor (BDNF) and tropomyosin-related kinase B (TrkB), particularly in the prefrontal cortex [51] and hippocampus [52], reversing dendritic spine loss in rodent models [53]. This pathway is particularly relevant to AD, where BDNF levels are significantly reduced, and genetic variations in BDNF are associated with increased depression risk [54, 55]. Nevertheless, the precise mechanisms underlying ketamine’s sustained antidepressant effects remain poorly understood, and the extent to which these mechanisms translate to therapeutic effects in neurodegenerative disease remains uncertain.

Clinically, ketamine appears to preferentially improve anhedonia, motivation, and cognitive symptoms [56–58] — domains often resistant to monoaminergic antidepressants [59]. A systematic review of cognitive outcomes reported improvements in processing speed, memory, and cognitive flexibility [60]. This pro-cognitive effect may relate to enhanced synaptic plasticity and hippocampal function, with imaging studies showing associations between hippocampal connectivity changes and treatment response [61, 62].

Anhedonia, defined as diminished interest or pleasure in rewarding activities, is common in PD and AD, affecting 40–60% of patients [63]. Dysfunction in reward-related brain circuitry and cognition play a central role in the development and severity of anhedonia and related motivational symptoms [64]. Imaging studies suggest that ketamine’s anti-anhedonic effects may be in part mediated by modulation of key regions within reward processing circuits, including normalisation of frontostriatal connectivity [65] and increased dorsal anterior cingulate activity [56, 66], changes that may enhance the flexible use of reward-related information and improve motivation.

Taken together, ketamine has robust evidence as a rapid-acting antidepressant in treatment-resistant depression, with effects on motivation and cognition and a coherent mechanistic rationale relevant to neurodegenerative disease. However, in the absence of disease-specific trials, its role in AD and PD remains untested.

##### Dextromethorphan

Originally marketed as a cough suppressant, dextromethorphan is an NMDA receptor antagonist with additional serotonin–noradrenaline reuptake inhibition. Repurposed for the treatment of pseudobulbar affect, it has since demonstrated clinical efficacy for agitation in AD [67]. In combination with the noradrenaline-dopamine reuptake inhibitor, bupropion, dextromethorphan was approved in the US in 2022 for the treatment of depression. This followed the phase III GEMINI trial (*n* = 327), in which twice daily treatment showed significant reductions in MADRS scores at six weeks (week 6 mean difference −3.4, 95% CI−5.5 to −1.4), with remission rates of 39.5 versus 17.3% for placebo [68].

There is currently no direct evidence supporting dextromethorphan’s antidepressant efficacy in AD or PD. However, in other neurodegenerative conditions, modest antidepressant effects of dextromethorphan have been observed in small studies in pseudobulbar affect [69] (week 12 Beck Depression Inventory-II (BDI-II) mean difference versus placebo: −1.6, SD 0.04) [69]. In AD, dextromethorphan-quinidine has demonstrated efficacy for agitation, where quinidine prolongs dextromethorphan’s effects by inhibiting hepatic metabolism [67]. Although depression was an exclusion criterion, secondary analyses reported small but statistically significant improvements in depressive symptoms (CSDD LS mean difference −1.3, 95% CI−2.6 to −0.1) [67].

Overall, while dextromethorphan is effective in major depressive disorder and is mechanistically plausible, with evidence of tolerability in AD, evidence for antidepressant efficacy in AD remains indirect, and no studies have been conducted in PD.

##### Esmethadone

Esmethadone, the opioid inactive (*S*)-enantiomer methadone, is a novel uncompetitive NMDAR antagonist with minimal mu-opioid affinity and no reported opioid effects, or abuse potential [70]. In a phase IIa trial in TRD (*n* = 62), once-daily esmethadone led to significant improvements in MADRS scores (LS mean difference −10.4; SMD 1.0) without dissociative or psychotomimetic effects [71]. However, a subsequent phase III trial was halted for futility, substantially weakening confidence in its efficacy. Although post-hoc analyses suggested potential benefit in specific antidepressant tolerant subgroups [72], development has since been abandoned, and no studies have evaluated esmethadone in neurodegenerative disease.

Preclinical data suggest that esmethadone’s potential antidepressant effects may involve induction of neurotrophic factors, including BDNF, which may be relevant to AD and PD where BDNF is reduced. However human evidence is limited to very small samples (*n* = 8) [73], and its relevance to neurodegenerative disease remains uncertain.

### Novel serotonin modulating therapies

#### The role of the serotonergic system in neurodegenerative disease and depression

The serotonergic system comprises multiple receptor subtypes (5-HT1–7) with region- and cell-specific effects and extensive modulation of other neurotransmitters, including dopamine. Converging evidence implicates complex receptor-specific dysfunction and dynamic interactions with other neurotransmitter systems in both neurodegenerative disease and depression.

Early neurodegeneration of the dorsal raphe nucleus (DRN), the principal source of cortical serotonergic projections, occurs in AD [74], with up to 77% of neuronal loss depending on disease stage [75]. Consequently DRN neurodegeneration is associated with reduced cortical serotonin, serotonin metabolites (5-hydroxyindoleacetic acid) and decreased serotonin transporter (SERT) levels [76, 77]. Disrupted serotonergic function in AD has been associated with disease progression and the emergence of behavioural and psychological symptoms, including depression. Post-mortem studies demonstrate that greater DRN neuronal loss and reduced cortical serotonin uptake sites correlate with depressive symptoms in AD [78, 79], while PET imaging shows reduced SERT binding associated with greater depressive severity [80].

In PD, DRN degeneration is comparable, although SERT binding may be preserved or even increased in depressed patients [81, 82]. PET imaging instead demonstrate reduced 5-HT1A receptor binding in the insula, hippocampus, and orbitofrontal cortex in PD depression [83], and serotonergic depletion within the DRN correlates with motivational symptom severity [84]. These findings suggest receptor- and circuit-specific serotonergic alterations rather than uniform depletion. While reduced 5-HT1A binding in limbic regions has been linked to depressive and cognitive symptoms, altered 5-HT2A expression relates more closely to hallucinations, and 5-HT4 receptor function, enriched in hippocampal circuits, may influence cognition. However, most evidence remains cross-sectional, and causal links between receptor alterations and treatment response are unclear. Clarifying these subtype-specific mechanisms represents an important priority for receptor-targeted serotonergic therapies.

Despite this mechanistic rationale, conventional selective serotonin reuptake inhibitors (SSRIs) show little evidence of efficacy for depression in AD or PD [85, 86]. Similarly, vortioxetine—a multimodal serotonergic agent (5-HT1A agonist; 5-HT1D/3/7 antagonist; SERT inhibitor)—improved mood and cognition in late-life depression [22], but failed to demonstrate benefit in a randomised trial (*n* = 100) in AD depression compared to placebo [87]. These findings suggest that broad serotonergic enhancement may be insufficient in the context of neurodegenerative pathology, supporting interest in receptor-selective approaches tailored to disorder-specific circuitry.

##### Prucalopride

Over 20 years ago, 5-HT4-receptor agonists were explored in AD but were limited by cardiovascular toxicity [88]. Prucalopride, a highly selective 5-HT4 receptor partial agonist with good brain penetration and established safety profile [89] approved for the treatment of constipation, has renewed interest in this pathway. Preclinical studies suggest that 5-HT4 receptor activation enhances hippocampal excitability [90], increases acetylcholine neurotransmission [89, 91], and reduces β-amyloid production via stimulation of α-cleavage of the amyloid precursor protein (APP) [92–94]. While prucalopride has not been evaluated as a treatment for depression in AD or PD, and its antidepressant potential is best considered exploratory at present, epidemiological studies report associations with reduced depression risk, alongside plausible effects on cognition and gut–brain pathways.

In humans, 5-HT4 receptor agonists enhance hippocampal-dependent memory processes [95], and reverse anticholinergic-induced cognitive impairment [95]. Short-term prucalopride administration increases hippocampal activation during memory tasks and improves cognitive performance in healthy participants [95, 96]. However, notably, an early PET study reported a negative association between hippocampal 5-HT4 binding and memory performance, underscoring the complexity of receptor–function relationships [97].

Large epidemiological studies suggest that prucalopride, in addition to its pro-cognitive effects, has possible antidepressant effects. Compared with other laxatives, prucalopride treatment was associated with reduced long-term depression risk (HR 0.79, 95% CI 0.69–0.91; *n* = 8281) [98]. Constipation is highly prevalent in PD (24–63%) [99], associated with depression risk (hazard ratio: 1.66) [100] and more severe in depressed PD patients (OR 5.81) [101]. While observational data cannot establish causality, these findings raise the possibility that 5-HT4 agonism may engage overlapping serotonergic and gut–brain mechanisms relevant to PD and AD depression. Clinical trials are needed, but prucalopride may represent an exploratory pro-cognitive candidate that warrants further investigation, with the additional benefit of addressing constipation.

##### Psychedelics

Psychedelics include naturally occurring and synthetic compounds such as psilocybin, ayahuasca, and lysergic acid diethylamide (LSD). Their primary psychoactive effects are mediated via 5-HT2A receptor agonism, with the exception of 5-MeO-DMT, which has higher affinity for 5-HT1A receptors and a shorter duration of action. Following early therapeutic exploration in the 1940s, research was halted in the 1970s after legal prohibition [102]. Renewed interest has led to multiple clinical trials in depression demonstrating rapid antidepressant effects, although concerns regarding functional unblinding remain, and no studies have examined psychedelics in patients with neurodegenerative diseases.

The largest published trial to date, a phase IIb RCT (*n* = 233), compared single-dose psilocybin (25 mg or 10 mg) with 1 mg control [103]. At week 3, MADRS reductions were −12.0 (25 mg), −7.9 (10 mg), and −5.4 (1 mg), with response rates of 36 versus 18% for high versus low dose. However, an earlier trial (*n* = 59) comparing two psilocybin sessions with escitalopram found no difference on the primary endpoint at week 6, despite positive secondary outcomes [104]. Smaller trials of ayahuasca (*n* = 29) [105] and dimethyltryptamine (DMT) (*n* = 10) [106], also reported rapid symptom reductions, but were limited by sample size and short follow-up.

Proposed antidepressant mechanisms include enhanced synaptic plasticity and neurotrophic signaling, increased global brain network integration, improved cognitive flexibility, and non-specific factors such as expectancy and unblinding [107, 108].

This is particularly relevant given marked alterations in 5-HT2A receptor expression in neuropsychiatric symptoms in AD and PD. PET studies in AD show reduced cortical and striatal 5-HT2A binding (Fig. 1), whereas PD is associated with increased cortical 5-HT2A expression linked to the emergence of hallucinations [109]. Increased 5-HT2A receptor expression and specific polymorphisms have also been consistently linked to psychotic symptoms in both AD and PD [110], and as 5-HT2A antagonism (pimavanserin) treats PD psychosis, psychedelic-induced hallucinations or delirium represent a key safety concern. Nevertheless, given preclinical and indirect evidence suggesting effects on synaptic plasticity and synaptogenesis, processes disrupted in PD and AD, further study is warranted [14]. Shorter-acting agents such as 5-MeO-DMT could offer a more pragmatic approach, though safety and tolerability in this population remain untested [111].

##### Pimavanserin

Despite being developed for PD, pimavanserin has not been evaluated for depression in PD or other neurodegenerative disorders, and negative phase III trials in major depressive disorder temper confidence in its antidepressant efficacy.

Pimavanserin, a selective 5-HT2A receptor inverse agonist approved for PD psychosis in 2016 [112], represents a mechanistically paradoxical strategy to psychedelics. Several antidepressants, including mirtazapine, and second-generation antipsychotics also bind 5-HT2A receptors, and cumulative evidence implicates this receptor in depression [113]. Cortical 5-HT2A receptors are highly co-expressed with 5-HT1A receptors, and 5-HT2A blockade may enhance limbic 5-HT1A-mediated neurotransmission, potentially improving affective and cognitive processing. PET imaging studies have revealed reduced 5-HT1A binding in temporal regions including the hippocampus and fusiform gyrus in depression [114–116].

In the phase II CLARITY trial (*n* = 207), adjunctive pimavanserin significantly improved 17-item Hamilton Depression Rating Scale (HDRS-17) scores at week 5 compared to placebo (mean change in HDRS-17: −4.0, *p* < 0.001) [117]. However, subsequent phase III trials were negative, and meta-analysis of two phase III RCTs found no clear efficacy [118].

### Dopaminergic therapies

#### The role of the dopaminergic system in neurodegenerative disease and depression

The dopaminergic system is central to goal-directed behaviour and learning by signalling deviations between expected and actual outcomes (“reward prediction error”), and by encoding reward value and effort costs of actions [119–121]. Disruption of these processes is most closely linked to motivational dimensions of depression, particularly anhedonia and apathy, which affect 40–60% of patients with PD or AD and form an “interest–activity” symptom cluster associated with poorer antidepressant response [59, 122]. These motivational symptoms, that share overlapping yet dissociable neurocognitive mechanisms, are partly distinct from affective symptoms such as low mood, guilt, or negative cognitions, which are more likely to respond to current antidepressant treatments.

The expression of dopaminergic dysfunction differs between disorders. In PD, dopaminergic neurodegeneration is profound, with up to 80% of striatal projections lost by diagnosis. PD patients report greater apathy and anhedonia in the “off” dopaminergic medication state [123, 124] and reduced striatal dopaminergic innervation correlates with motivational, but not global, depressive symptoms [125]. Behavioural paradigms support this distinction. Depressed PD patients completing an effort-based decision-making tasks demonstrate reduced reward sensitivity and diminished willingness to exert effort for reward that correlates with motivational symptom severity, particularly in the dopaminergic “off” state.

These findings support a model in which dopaminergic dysfunction disrupts reward processing and effort allocation, giving rise to motivational depressive symptoms, particularly in PD.

Clinical data align with this dimensional framework. In a longitudinal cohort of over 400 PD patients, dopamine agonists were associated with improvements in motivational symptoms, while monoamine oxidase-B (MAO-B) inhibitors improved both depressive and motivation symptoms [126].

In AD, dopaminergic changes are less severe and more heterogeneous, with mesocorticolimbic disruption occurring alongside broader cortical and cholinergic dysfunction. Apathy has been linked to disruption of mesocorticolimbic dopaminergic and noradrenergic signalling in the prefrontal cortex, where motivational deficits correlate with amyloid burden. This has informed the repurposing of medications such as methylphenidate, which may improve motivation by enhancing dopamine and noradrenaline transmission within prefrontal–striatal circuits [127].

Together, these findings suggest that dopaminergic dysfunction plays a more central and tractable role in depression in PD, whereas in AD it represents one component of a broader network disturbance. Accordingly, behavioural and neurobiological markers, such as effort-based decision-making parameters and striatal dopaminergic integrity, may be more useful for guiding treatment in PD than in AD, where treatment response may be less closely linked to dopaminergic dysfunction.

##### Dopamine agonists

Pramipexole, a selective D2/3 receptor agonist, is among the few non-serotonergic therapies evaluated for depression in depression in neurodegenerative disease. In an RCT of 287 patients with PD depression, pramipexole significantly improved depressive symptoms at 12 weeks (mean difference on Beck Depression Inventory: 1.9, 95% CI 0.5–3.4), independent of motor improvement [128]. Trials in patients with depression without neurodegenerative disease have also shown that pramipexole is associated with superior response rates compared with placebo (RR: 1.77; 95% CI: 1.11–2.82) [129–131]. More recently, a 48-week trial in treatment-resistant depression (*n* = 151) demonstrated a 44% response rate, 28% remission rate, and a large effect size relative to placebo (SMD 0.87) [132].

Mechanistically, pramipexole acts primarily via D2/3 receptor activation. D3 receptor expression is localised within the mesolimbic system, particularly the ventral striatum—a key node in reward processing [133]. In healthy participants pramipexole enhances reward learning by preserving learned values and accuracy of choices during reward based learning tasks [134, 135], providing a plausible cognitive mechanism underlying its pro-motivational and antidepressant effects. However, an important clinical consideration with dopamine agonists, particularly in PD, is the risk of impulse control disorders (ICDs). Dopamine agonists are well established to increase vulnerability to ICD behaviours such as pathological gambling, hypersexuality and compulsive shopping, likely reflecting overstimulation of mesolimbic reward circuits. Thus, potential benefits in depression must be balanced against behavioural risks, necessitating careful selection, monitoring, and dose titration.

Dopaminergic dysfunction is also evident in AD. Approximately 40% of AD cases show pathological involvement of ventral tegmental area (VTA) projections to the ventral striatum, including amyloid-β deposition, neurofibrillary tangles, and reduced dopamine synthesis. Despite this, pramipexole has not been evaluated in AD depression.

Other dopaminergic agents have been explored. In a large RCT (*n* = 200) in AD, methylphenidate, a dopamine–noradrenaline reuptake inhibitor, demonstrated modest but statistically significant improvement in apathy (NPI apathy mean difference −1.25; 95% CI−2.03 to −0.47; *p* = 0.002) [136]. However, patients with depression were excluded, and no change in depressive symptoms was observed. Thus, while dopaminergic augmentation may improve motivational deficits in AD, evidence for antidepressant efficacy in this population remains limited.

### KCNQ potassium (K+) channel modulators

Seizures are common in neurodegenerative disease, particularly AD where they occur in 10–22% of patients, rising to 64% in longitudinal cohorts across disease stages [137]. PD is also associated with elevated seizure risk (OR 1.68; 95% CI 1.43–1.98) compared to age-matched controls [138].

In AD, seizures are linked to earlier onset and accelerated cognitive decline. Subclinical epileptiform activity—particularly in frontotemporal regions—is common and has been linked to BPSD, including irritability and affective disturbance [139]. EEG abnormalities are also well documented in depression [140], and predict poorer response to antidepressants [141]. Alongside the use of antiepileptics such as lamotrigine in TRD, these findings suggest that dysregulated neuronal excitability may contribute to depressive symptoms in neurodegenerative disease and represent a therapeutic target.

KCNQ2/3 channels are voltage-gated potassium channels widely expressed in hippocampal and cortical regions that regulate neuronal excitability. In preclinical studies KCNQ2/3 activation has been shown in preclinical models to reduce neuronal hyperexcitability and restore phasic dopaminergic firing from the ventral tegmental area, reducing anhedonic behaviours [142]. This mechanistic rationale has prompted clinical evaluation of KCNQ channel openers in depression.

#### Ezogabine

Ezogabine is a selective KCNQ potassium channel opener that enhances transmembrane potassium currents, reducing neuronal excitability. Originally developed as an antiepileptic, it has also been studied in motor neuron disease due to its ability to reduce cortical hyperexcitability [143].

Early-phase studies in depression suggest potential antidepressant effects. An initial open-label study in depression reported significant improvement in anhedonia, alongside enhanced reward learning and increased ventral striatum–cingulate connectivity [144]. A subsequent RCT in depression, (*n* = 45) showed greater symptom improvement versus placebo, with a large effect size (Cohen’s d = 0.76 for change in MADRS) [145]. Anhedonia improved significantly (mean difference −6.9 on SHAPS), and fMRI analyses indicated that clinical response was associated with increased VS activation during reward anticipation [145]. These findings are consistent with enhanced reward sensitivity as a potential therapeutic mechanism. Given the role of reduced reward sensitivity in PD depression, KCNQ modulation is theoretically relevant [21, 146]. However, ezogabine has not been studied in AD or PD and available clinical data are limited to small samples. Collectively, while mechanistically compelling for PD depression, evidence for antidepressant efficacy remains preliminary.

### Kappa opioid receptor therapies

Kappa opioid receptors (KOR) and their endogenous ligand, dynorphin, are expressed in key stress and reward-related regions, including the amygdala, striatum, and anterior cingulate cortex [147, 148]. KOR activation inhibits mesolimbic dopamine release, contributing to dysphoria and aversive learning [149]. Preclinical studies indicate that stress-induced dynorphin overactivation of the KOR system may contribute to anhedonia, and that selective KOR antagonism may reverse this effect [150, 151] by disinhibiting dopaminergic signalling within reward circuits [149].

Alterations in the KOR system have been reported in neurodegenerative disease [152]. Post-mortem studies in AD have shown significant increases in striatal KOR binding, and elevated DNA methylation at the KOR promoter compared to controls [153, 154]. Given the prominence of motivational symptoms and dopaminergic dysfunction in AD and PD, KOR modulation represents a plausible target.

#### Buprenorphine

Buprenorphine is a partial mu-opioid receptor agonist and KOR antagonist has been studied in combination with the mu-antagonist samidorphan, to reduce abuse potential. Two early RCTs showed superiority over placebo [155], but three additional trials were negative, and a 2020 meta-analysis found no significant benefit (*n* = 1242, SMD = −0.07, 95% CI: −0.21 to 0.06) [155]. Given the risks associated with opioid agonism in older adults, more selective novel KOR antagonists may represent a more promising strategy.

#### Aticaprant/Navicaprant

In a phase II trial of adjunctive aticaprant (a selective KOR antagonist) (*n* = 169) in patients receiving SSRI/SNRI therapy, once-daily dosing was associated with significant improvement in depressive symptoms at week 6 (LS mean MADRS difference −3.1; upper limit 1-sided 80% CI 2.21) and reduced anhedonia [148].

However, the 2025 phase III KOASTAL-1 trial of navicaprant failed to demonstrate improvement over placebo in depression [156]. This negative outcome substantially tempers confidence in KOR antagonism as a broadly effective antidepressant strategy, and no trials currently ongoing for depression.

### γ-amino butyric acid (GABA) modulators

The GABAergic system, comprising ionotropic (GABAA, GABAC) and metabotropic (GABAB) receptors, maintains excitatory–inhibitory balance and regulates learning, memory, and attention. Disruption has been implicated in the aetiology of both depression and neurodegeneration.

In AD, amyloid precursor protein interacts with GABA_B_ receptors, reducing synaptic GABA signalling, driving degeneration of GABAergic interneurons in the perirhinal cortex [157, 158].

GABAergic dysfunction has also been reported in depression. Plasma and cortical GABA levels are reduced and correlate with anhedonia severity, particularly in the anterior cingulate cortex [159, 160]. Benzodiazepines, positive allosteric modulators of GABA_A_ receptors, can provide rapid anxiolytic and short-term mood benefits [161], but are limited by tolerance, dependence, falls, cognitive impairment, and increased mortality in older adults [162]. This has driven the development of novel GABA-targeting therapies.

#### Brexanolone

Brexanolone, an intravenous formulation of neurosteroid allopregnanolone, and positive GABA_A_ modulator. restores excitatory–inhibitory balance and mimics endogenous neurosteroid fluctuations. Brexanolone was approved by the FDA in 2019 for postpartum depression (PPD) following three RCTs. Pooled analysis of two of these trials (*n* = 138) showed significant improvement in depressive symptoms after a single 60-h infusion (HAM-D LS mean difference −3.7; 95% CI−6.9 to −0.5), with effects sustained to day 30 [163, 164].

Subsequently, zuranolone an oral analogue, was approved by the FDA in 2023 for PPD and has shown promising results for MDD [165]. In a phase II trial (*n* = 89), once-daily zuranolone significantly reduced depressive symptoms by day 15 (HAM-D LS mean difference −7.0; 95% CI−10.2 to −3.9) [166]. However, the FDA declined approval for depression due to insufficient evidence of durable efficacy, underscoring uncertainty regarding long-term benefit [165, 167].

To date, neither agent has been studied in patients with AD or PD. Preclinical data suggest allopregnanolone reduces amyloid-β deposition and microglial activation in AD models and promotes neuroprotective protein expression in PD models [168, 169]. While mechanistically promising, more consistent clinical evidence in depression is needed before translation to neurodegenerative populations.

## Immune modulating/anti-inflammatory agents

Inflammation and immune dysregulation have long been hypothesised to be key mechanisms underlying neurodegenerative disease [170]. In AD microglial activation is an early and potentially initiating process, implicated in the development of amyloid pathology and linked to genetic risk, with longitudinal evidence suggesting a dynamic shift from protective to deleterious effects as disease progresses [171]. In PD, inflammatory changes are also observed, including activated midbrain microglia [170, 172], with PET studies linking microglial activation to cognitive decline but not disease severity [173, 174]. Elevated pro-inflammatory cytokines, particularly tumour necrosis factor (TNF), are reported in serum, CSF, and brain tissue in PD [175]. In animal models, TNF blockade attenuates dopaminergic neurodegeneration [176] and retrospective cohorts receiving anti-TNF therapy for inflammatory bowel disease show reduced PD incidence, though these findings have not translated into disease-modifying treatments [177]. In contrast, meta-analyses of peripheral pro-inflammatory cytokines have shown no difference in AD, suggesting that central inflammatory processes are more prominent in the condition [178].

Low-grade inflammation is well established in depression [179]. Approximately one-third of patients exhibit elevated C-reactive protein (>3 mg/L) [180], alongside increased interleukin-6 (IL-6) and other cytokines in serum and CSF. Perhaps most compellingly, immune activation via interferon-α treatment for hepatitis C induces depression in up to half of patients [181]. Mendelian randomisation studies support a potential causal role for IL-6 in specific depressive symptoms, although CRP associations may be partly mediated by metabolic factors [182].

In PD depression, evidence linking inflammation to depression remains largely peripheral and associative. A recent systematic review identified 14 studies examining inflammatory markers in PD depression, most of which were cross-sectional, with elevations in CRP and TNF-α reported inconsistently [183]. In contrast, multimodal PET imaging in AD demonstrates a more direct relationship between central neuroinflammation and neuropsychiatric symptoms, with microglial activation in frontal, temporal, and default mode network regions associated with symptom severity independently of amyloid and tau pathology [184].

These findings suggest that inflammatory mechanisms in AD are more centrally mediated, with microglial activation closely linked to cortical network dysfunction and neuropsychiatric symptom expression. In contrast, in PD, inflammatory changes appear more peripheral and secondary, reflected in circulating cytokine alterations that are less clearly tied to symptom generation. This distinction may have therapeutic implications, with centrally acting or microglial-targeted approaches potentially more relevant in AD, whereas peripheral anti-inflammatory strategies in PD may be less likely to directly modify depressive symptoms. However, no trial has specifically targeted patients with depression in neurodegenerative disease, and to date, trials of anti-inflammatory therapies in depression have largely produced modest or negative results.

### Non-steroidal anti-inflammatories

Several trials of cyclooxygenase (COX) inhibitors, including aspirin and celecoxib, have been evaluated in depression. A meta-analysis of 26 RCTs found moderate symptom reduction ((SMD −0.55, 95% CI −0.75 to −0.35) compared to placebo [185], but high-quality trials have generally failed to confirm clinically meaningful benefit. A 12-week RCT of celecoxib (*n* = 66) found no superiority over placebo [186], and a large double-blind trial of low-dose aspirin (*n* = 19,114) demonstrated no preventive effect and increased depressive symptoms with prolonged use [187]. Overall, evidence for NSAIDs as antidepressants is weak; despite longstanding clinical use, they have not demonstrated clinical clear efficacy in depression.

### Minocycline

Minocycline is a tetracycline antibiotic that crosses the blood-brain barrier, inhibits microglial activation, and modulates GABA signalling. Although initially proposed as a disease-modifying therapy in AD, large trials were negative [188]. In depression, early small studies suggested benefit. A meta-analysis of three trials (*n* = 151) reported a significant reduction in depressive symptoms with adjunctive minocycline (SMD−0.79; 95% CI−1.29 to −0.28; *p* = 0.002). However, subsequent larger RCTs have been negative. A 12-week multi-arm trial (*n* = 266) found no significant improvement in HAMD-17 scores [186], and a more recent trial of 173 patients with depression also found no difference from placebo at 6 weeks [189]. These results substantially weaken support for minocycline as an antidepressant, although subgroup analyses suggest possible benefit in depressed patients with low grade inflammation.

### Anti-cytokine monoclonal antibodies

Despite the limited success of recent immune-modulating anti-inflammatory therapies, there remains optimism about the future potential of this therapeutic class. A meta-analysis of anti-TNF agents across five RCTs reported small but significant improvements in depressive symptoms (SMD 0.33; 95% CI 0.06–0.60) [190]. These findings are consistent with a small RCT in TRD patients suggested benefit from infliximab, particularly in those with high baseline inflammation [191]. However, larger subsequent trials in bipolar depression were negative [192], and a small RCT of etanercept in AD (*n* = 41) found no effect on cognition or depressive symptoms [193]. In response, trial designs incorporating enrichment strategies based on baseline inflammatory status have been proposed. Trials using this approach are underway, IL-6 inhibition with tocilizumab [194].

**Fig. 3:**
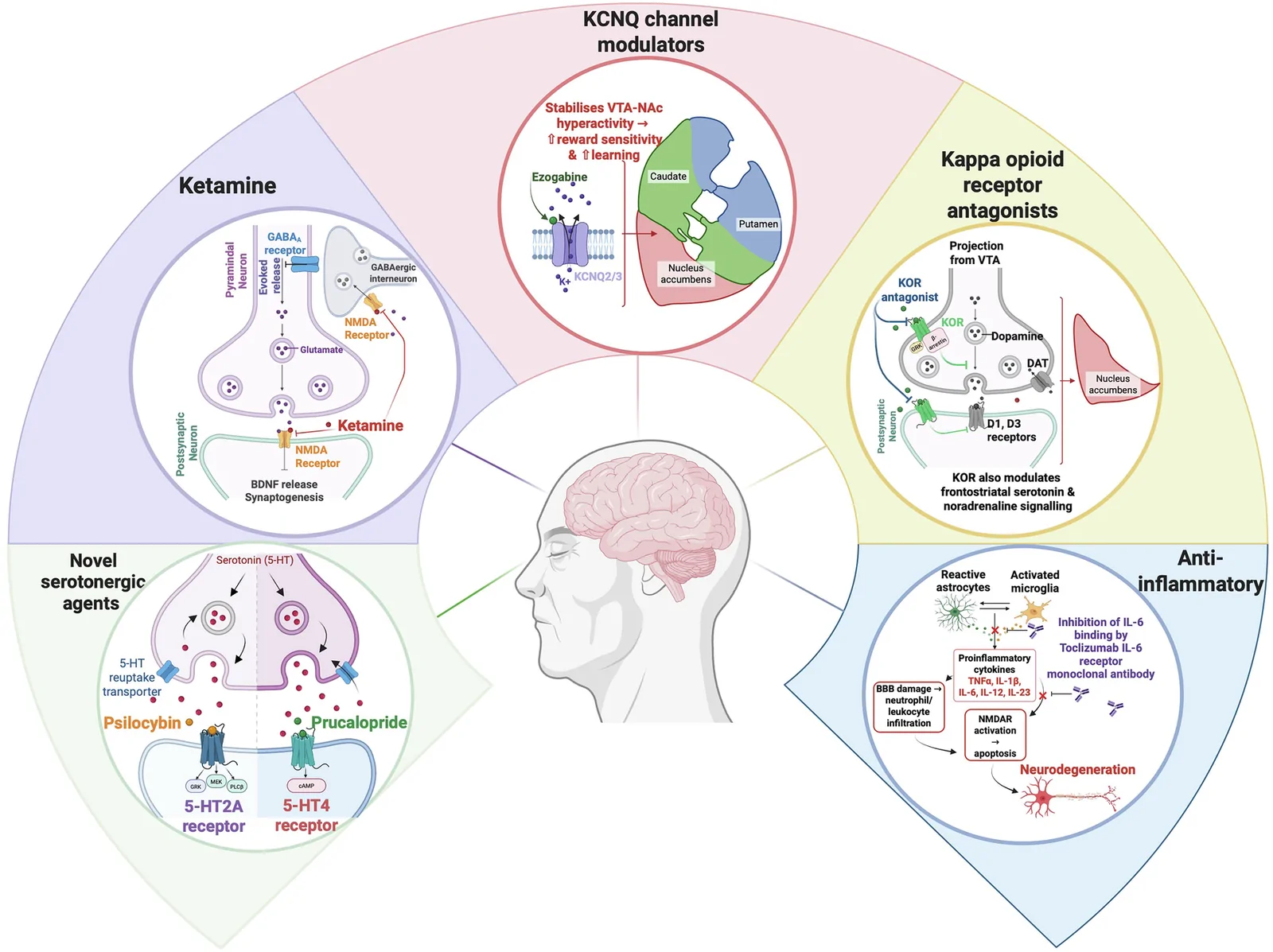
Mechanistic targets of emerging pharmacological therapies for depression in neurodegenerative disease. Schematic overview of putative mechanisms of action for selected novel or repurposed therapies discussed in this review. Interventions are organised by primary mechanistic domain: glutamatergic modulation (ketamine), serotonergic receptor–specific agents (e.g., 5-HT2A agonists, 5-HT4 agonists), KCNQ channel modulators (stabilising mesolimbic dopaminergic hyperactivity), kappa opioid receptor (KOR) antagonists (modulating mesostriatal dopamine signalling), and anti-inflammatory agents (targeting cytokine-mediated neuroinflammation and blood brain barrier disruption (BBB)).

Taken together, several promising pharmacological strategies have emerged that move beyond conventional monoaminergic paradigms and offer potential for disease-specific application in neurodegenerative conditions (see Table 1 for summary of most promising candidates). These agents—spanning glutamatergic modulators, receptor-selective serotonergic compounds, dopaminergic and kappa opioid–targeting therapies, KCNQ channel modulators, and immune-directed interventions—reflect a shift toward mechanistically informed treatment development. As summarised in Fig. 3, although pharmacologically distinct, emerging therapies converge on overlapping circuit-level processes implicated in depression in neurodegenerative disease, including reward valuation, synaptic plasticity, and inflammatory modulation. This convergence supports a framework in which treatments are aligned with disorder-specific molecular and network pathology in AD and PD, rather than extrapolated from younger populations.

**Table 1:** Summary of promising novel pharmacological therapies for depression in neurodegenerative disease.

Drug	Class	Proposed Mechanism of Action	Effect size in MDE	Positive Phase III Trial in MDE?	Tested in AD or PD?	Side Effect Burden	Pro-Cognitive Effects	Pro-Motivational	Additional benefits
Ketamine / Esketamine	Glutamatergic	NMDAR antagonist	Large	Yes	No	Moderate (dissociation, blood pressure changes, risk of psychosis)	Yes	Yes	-
Dextromethorphan		NMDAR antagonist	Small	Yes	Only agitation in AD	Low to Moderate	Unknown	Likely	-
Esmethadone		NMDAR antagonist	Large (phase II)	No (trial halted)	No	Low	Potential	Likely	-
Pramipexole	Dopaminergic	D2/D3 dopamine agonist	Large	Yes	Yes (PD)	Moderate (impulse control, nausea)	Potential	Yes	Improves motor symptoms
Methylphenidate		Dopamine/noradrenaline reuptake inhibitor	Unknown	No	Yes (AD)	Moderate (BP, appetite, sleep)	Yes	Yes	Improves concentration
Psilocybin	Serotonergic	5-HT2AR agonist (psychedelic)	Moderate	No (only Phase IIb, phase III underway)	No	Moderate to High (hallucinations, psychosis)	Potential	Yes	-
Prucalopride		5-HT4R partial agonist	Unknown	No	No	Low	Yes	Unknown	Treats constipation
Pimavanserin		5-HT2AR antagonist/inverse agonist	Small	No	Approved for PD psychosis only	Low	Unclear	Possible	Antipsychotic
Brexanolone / Zuranolone	GABAergic	GABAA receptor modulator	Moderate	Yes (postpartum depression)	No	Moderate (sedation, somnolence)	Potential	Unknown	-
Ezogabine	Other	KCNQ potassium channel opener	Moderate	No	No	Moderate	Unknown	Yes	Anti-seizure
Aticaprant / Navicaprant		Kappa opioid receptor antagonists	Low to Moderate	No (Navicaprant failed)	No	Low to Moderate	Unknown	Yes	-
Minocycline	Anti-inflammatory	Anti-inflammatory + GABA modulation	Moderate	No	Yes (negative in AD)	Low	Potential	Unknown	-
Tocilizumab / Infliximab		Monoclonal antibodies (IL-6, TNF)	Unknown	No	No	Moderate to High	Unknown	Unknown	-

(*AD* Alzheimer’s disease, *PD* Parkinson’s disease, *MDE* major depressive episode, *NMDAR* N-methyl-D-aspartate receptor, *5-HT2AR / 5-HT4R* 5-hydroxytryptamine (serotonin) receptor subtype, *GABAA* gamma-aminobutyric acid type A receptor, *KCNQ* voltage-gated potassium channel subfamily Q, *TNF* tumour necrosis factor, *IL-6* Interleukin-6).

## Brain stimulation

Over the past two decades, evidence and methods of neuromodulation in depression has expanded substantially. Although often discussed as discrete interventions, neuromodulation modalities can be viewed as complementary tools for targeting distributed depression-relevant networks that are disrupted in AD and PD.

Functional connectivity studies link depression in neurodegenerative disease to limbic–prefrontal and frontostriatal disruption. In AD, depressive symptoms are associated with reduced amygdala–default mode connectivity and weakened prefrontal–medial temporal coupling, suggesting impaired top-down regulation of affective circuits [195]. In PD, depression severity correlates with basal ganglia dysfunction and altered frontoparietal, reward, and salience network connectivity [196, 197]. Together, these findings suggest a rationale for individualised or combined strategies using cortical stimulation approaches, alongside novel methods, such as transcranial focused ultrasound (TUS), capable of precisely targetting subcortical drivers of network dysfunction.

### Electroconvulsive therapy (ECT)

Despite advances, electroconvulsive therapy (ECT) remains the most effective intervention for severe TRD, including in older adults and those with neurodegenerative disease [198]. A meta-analysis in PD reported large improvements in both motor symptoms (13 studies, *n* = 116; SMD = 1.18) and depression (6 studies, *n* = 51; SMD = 1.33), without evidence of cognitive decline [199]. Similarly, in AD, multiple case series and cohort studies have shown consistent benefits of ECT for depression [200], and RCTs demonstrate safety and efficacy for AD agitation [200]. Although no RCT has examined ECT specifically for depression in neurodegenerative disease, available evidence supports efficacy.

Mechanisms remain unclear, but ECT has been associated with increased striatal D1/D3 binding in animals and elevated CSF dopamine metabolites in humans, suggesting possible modulation of dopaminergic and reward-processing systems [201, 202].

Despite this evidence of efficacy and advocacy for wider access to ECT in neurodegenerative disease, its use in this population remains limited [203], likely due to concerns about cognitive side effects—despite no evidence of long-term impairment in late-life depression [204]— and service barriers [205].

While newer techniques may appear more acceptable and less stigmatised interventions for both patients and clinicians, ECT remains an important treatment option for severe, refractory cases in this population.

### Repetitive transcranial magnetic stimulation

Repetitive transcranial magnetic stimulation (rTMS) is a non-invasive neuromodulatory treatment that has well-established efficacy in the management of depression. It involves the delivery of electromagnetic pulses to modulate cortical excitability, typically via high-frequency stimulation of the left dorsolateral prefrontal cortex (DLPFC) and low-frequency right DLPFC stimulation [206]. Although these protocols exert opposing effects, their clinical efficacy appears comparable [207].

A 2022 meta-analysis of 14 RCTs in older adults reported significant improvements in depressive symptoms (SMD = 0.36; 95% CI 0.13–0.60) and remission rates (OR = 4.63; 95% CI 2.24–9.55) [208]. However, earlier analyses suggest reduced efficacy in individuals aged over 65 [209].

Standard rTMS therapy consists of once daily 30–45-min sessions, over a minimum of four weeks. However, accelerated protocols over once week, such as Stanford Neuromodulation Therapy protocol have demonstrated comparable efficacy to standard regimens in small trials [210].

In PD, a meta-analysis of seven double-blind RCTs found modest benefit versus sham (SMD = 0.42; 95% CI 0.13–0.70) [211]. However, included studies were small (largest RCT *n* = 44), used heterogeneous protocols, and trials comparing rTMS to antidepressants showed no superiority [206]. In AD, pooled analysis of two small studies (combined *n* = 71) showed no significant effect on depressive symptoms (SMD = −1.36; 95% CI−3.93 to 1.21) [212].

While further trials are needed to evaluate the effectiveness of rTMS for depression in neurodegenerative disorders, A key limitation of rTMS is shallow penetration (~2 cm), restricting modulation of subcortical structures implicated early in PD and AD [213].

### Transcranial direct current stimulation

Transcranial direct current stimulation (tDCS) delivers low-intensity electrical stimulation via scalp electrodes that can be self-administered at home. A meta-analysis of 9 trials for depression (*n* = 572) showed superiority over sham for response (OR = 1.96; NNT = 9) and remission (OR = 1.94; NNT = 13) [214]. A subsequent larger RCT (*n* = 174) of home-based tDCS demonstrated small but significant improvement (HDRS mean difference 2.26; Cohen’s d = 0.37) [215]. A recent large RCT combining tDCS with cognitive remediation reported modest slowing of cognitive decline over several years in older adults with remitted depression and mild cognitive impairment, but no effect on MADRS scores [216]. Although meta-analyses of other small studies in AD and PD report cognitive benefit, no trial has evaluated tDCS for depression in this population [217, 218]. The feasibility of home delivery may offer a practical and scalable neuromodulatory approach for patients with AD and PD with more advanced disease that warrants further study.

### Transcranial focused ultrasound

Recent advances enabling the non-invasive modulation of highly localised deep brain structures have led to a surge of interest in the use of TUS [219] Ablative TUS subthalamotomy is an established treatment for PD motor symptoms [220]. Low-intensity TUS has more recently been shown to modulate subcortical circuits without ablation, including reward-related regions, potentially enabling targeted stimulation of key subcortical regions implicated in depression in neurodegenerative diseases. Preclinical primate studies show that TUS targeting the lateral orbitofrontal cortex disrupts reward learning [221], while medial PFC stimulation reduces dopamine release in the ventral striatum [222]. Additional work in primates shows that dorsal striatal TUS increases reward motivation [223], and a recent study in healthy human participants demonstrated modulation of reward sensitivity following ventral striatal stimulation [224], providing proof of concept for precise modulation of reward circuitry implicated in PD depression.

Clinical evidence in depression is early-phase. A small RCT (*n* = 19) targeting the subcallosal cingulate showed symptom improvement at 24 h (Cohen’s d = −1.08; *p* = 0.031) but not at 7 days versus sham [225]. TUS offers a potentially unique option for non-invasive, precise modulation of subcortical networks implicated in reward and motivation, though evidence remains preliminary

### Deep brain stimulation

Approximately 5% of PD patients are candidates for deep brain stimulation (DBS) for motor symptoms [226]. Potential targets for PD depression include regions such as the VS known to play a crucial role in reward processing, motivation and mood. Trials of DBS for treatment-resistant depression outside PD have largely been negative [227], though secondary analyses and adaptive “closed-loop” systems show promise [228, 229], and VS DBS for OCD has demonstrated clear mood improvements preceding symptom relief [230, 231].

DBS has not been tested in dedicated trials for PD depression, but may represent a rational option in patients already receiving DBS or with severe treatment resistance.

## Challenges and opportunities in the development of novel therapies for depression in neurodegenerative disease

*‘To have success in research one needs the four ‘Gs’: Geld (‘money’), Geduld (‘patience’), Geschick (‘skills’) and Glück (‘luck’)’ – Paul Ehrlich (1854–1915), Nobel prize-winning physician and scientist who developed a cure for syphilis and invented Gram staining*.

This review has examined the neurobiological mechanisms underpinning depression in AD and PD, and evaluated emerging pharmacological and neuromodulatory therapies targeting disease-specific circuit, cognitive, and molecular dysfunction. Across therapeutic domains, a consistent pattern emerges: depression in neurodegenerative disease is mechanistically distinct from depression in the general population, yet clinical trials have largely failed to reflect this distinction. The consequence has been a persistent translational gap. Despite advances in neuroscience, imaging, and novel antidepressant development, patients with depression in AD and PD remain relatively neglected with a lack of effective therapies. Our review challenges the implicit assumption that depression in AD and PD is simply “comorbid depression”, and instead argues that it reflects disease-specific mechanisms that require targeted therapeutic approaches—and represent an opportunity for mechanistically informed innovation.

One of the greatest challenges in developing novel and effective therapies for depression in neurodegenerative disease has been the relative lack of Ehrlich’s ‘four Gs’. Few large, high-quality trials of novel treatments have been conducted in this population. Several structural and methodological factors likely contribute to this gap. First, depression is frequently listed as an exclusion criterion in disease-modifying trials for AD and PD. Even when present, depressive symptoms are rarely assessed as primary outcomes. For example, monoclonal anti-amyloid and anti-alpha-synuclein therapies have reported depression only as an adverse event, not as a therapeutic target. This persistent separation between “neurological” and “psychiatric” outcomes reflects a lingering mind–body dualism that fragments care and impedes translational progress. This missed opportunity is despite notable examples of neurological therapies being successfully repurposed for mood disorders (e.g., lamotrigine, pramipexole, pregabalin), thereby missing the opportunity to harness one of the most powerful drivers of psychiatric drug discovery: serendipity.

A second barrier is insufficient biological stratification. Many antidepressant trials in older adults enrol heterogeneous samples without phenotypic or biomarker enrichment. Immune-modulating agents have shown mixed results in unselected populations, yet stratification based on inflammatory markers such as CRP or IL-6 is only now being implemented. Similarly, dopaminergic agents may preferentially benefit patients with prominent motivational symptoms, but global rating scales dilute these dimension-specific effects. Without alignment between mechanism and phenotype, therapeutic signal can be obscured.

Heterogeneity of depression continues to pose a significant challenge for the field. While depression in the context of neurodegenerative disease may represent a more homogeneous clinical entity—offering clearer insights into its temporal onset and associated disease markers compared to depression in the general population—it remains a moving target. As neurodegenerative diseases progress and more brain systems become affected, the nature of depressive symptoms can shift. For instance, affective symptoms in dementia exhibit considerable variability, both between and within individuals over time. These symptoms may gradually evolve into an amotivated or apathetic state as cognitive impairment worsens [232]. Treating depression in this context therefore requires recognition of its dynamic trajectory rather than assuming static symptom constructs.

Age-related pharmacokinetic and pharmacodynamic factors further complicate treatment and alter the balance between tolerability and therapeutic efficacy. Reduced absorption, hepatic metabolism, renal clearance, and polypharmacy increase variability in drug exposure. Less appreciated, but potentially crucial, are disease-related changes in blood–brain barrier permeability, transporter expression, endogenous neurotransmitter release, and receptor reserve [233]. Reduced receptor reserve may alter the functional consequences of receptor occupancy, meaning that standard dosing paradigms derived from younger populations may not translate to neurodegenerative contexts. This has been demonstrated in the context of antipsychotic drug use [234, 235], but has been relatively neglected in relation to treatments for depression in the context of neurodegenerative diseases.

**Fig. 4:**
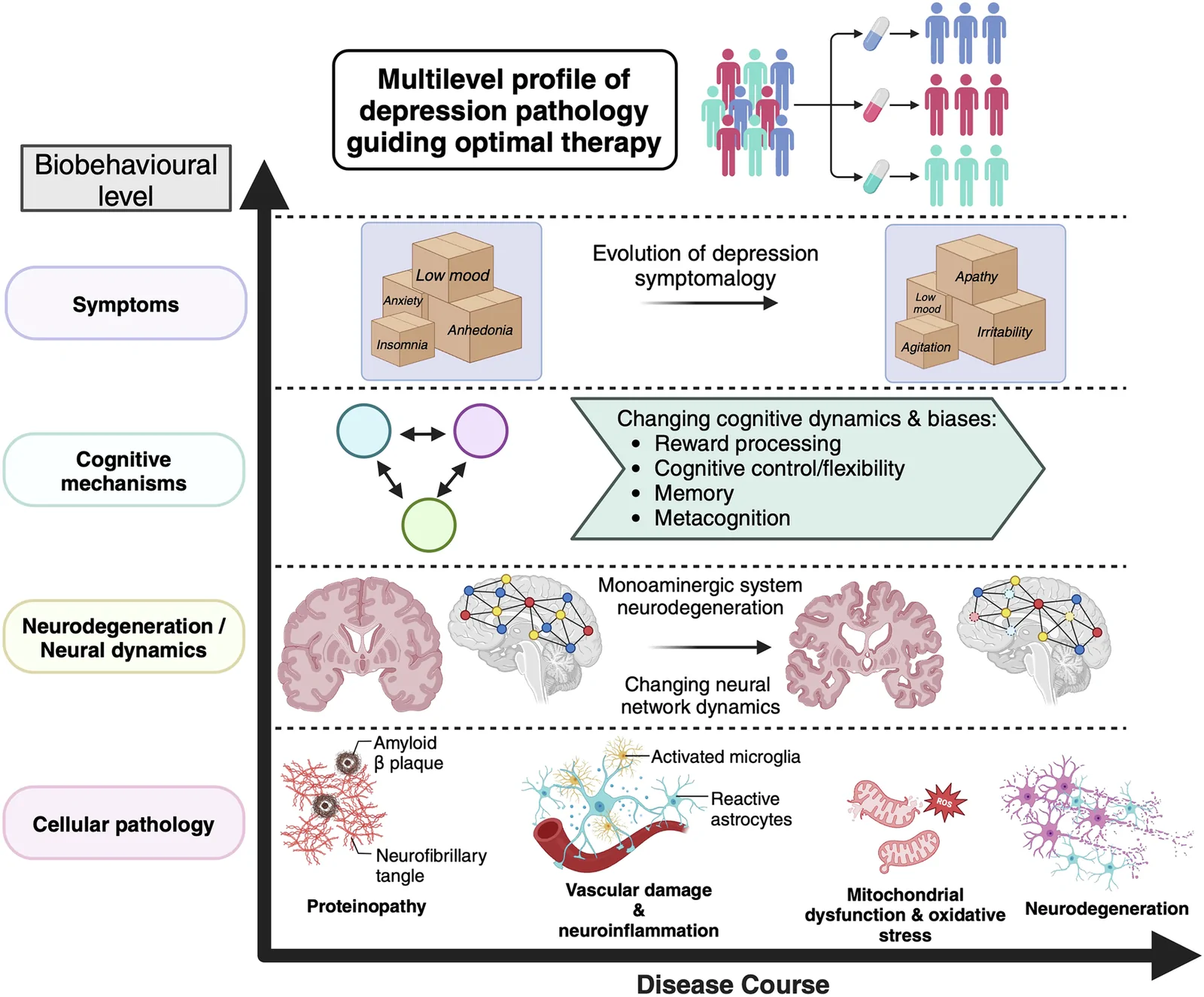
Multilevel mechanistic model of depressive pathology in neurodegenerative disease. Conceptual framework illustrating how cellular pathology (e.g., proteinopathy, neuroinflammation, mitochondrial dysfunction) interacts with neural network disruption and evolving cognitive mechanisms to shape depressive symptoms across disease progression.

Yet these challenges also reveal opportunity. Depression in the context of neurodegenerative disease offers a uniquely tractable model for mechanistically guided antidepressant development. Few clinical populations allow simultaneous mapping of molecular pathology, circuit dysfunction, behavioural phenotype, and longitudinal disease progression. In AD and PD, we can leverage existing understanding and cohort data on blood-based biomarkers, PET imaging of monoaminergic systems, inflammatory indices, cognitive paradigms, and functional connectivity measures that can then be integrated to define biologically informed subgroups and guide targeted treatment strategies, as shown in Fig. 4.

This multilevel framework (see Fig. 4.) provides a structured model linking molecular, circuit, and behavioural processes, and aligns with emerging precision psychiatry approaches that favour stratified or enrichment-based trial designs over broad diagnostic categories. There are growing calls to develop a transdiagnostic taxonomy of psychotropic medications grounded in neurobiological and psychopathological markers [236]. Depression in neurodegenerative disease may offer a uniquely informative setting for antidepressant development, as pathology is partially observable, disease progression is longitudinally staged, and symptom dimensions can be linked to specific circuit dysfunction. For example, integrating blood-based biomarkers such as pTau217 with cognitive and behavioural markers of depression in AD may enable tracking of the evolving mechanisms underlying depressive symptoms and support stratification of patients in trials of novel therapies or earlier intervention. Dopaminergic agents could be preferentially evaluated in patients with demonstrable striatal dysfunction, impaired reward processing, and prominent motivational symptoms, while combined PET imaging markers of central inflammation and peripheral cytokine profiles could help identify patients most likely to benefit from specific immune-modulating therapies and at which stage of illness. Multilevel integration of neurocognitive mechanisms with symptom profiles may also inform more precise targeting of neuromodulatory interventions, such as ventral striatal focused ultrasound in PD patients with frontostriatal circuit dysfunction during reward processing that is unresponsive to other treatments.

Crucially, this framework must also incorporate safety and tolerability as a central translational filter rather than a secondary consideration. Interventions with psychotomimetic effects, cognitive burden, or complex administration requirements may therefore have limited real-world applicability, even where mechanistic rationale and efficacy signals are strong (see Table 1 for summary of side effect burden of promising candidate therapies). Accordingly, evaluation of emerging therapies should integrate tolerability, cognitive effects, interaction with existing treatments, and caregiver feasibility alongside efficacy, with particular caution in populations at risk of psychosis with treatments such as ketamine, dopamine agonists and psychedelics. Viewed in this context, therapeutic promise in AD or PD depression is defined not only by biological plausibility, but by patient-centred safety and usability, and highlights an opportunity to develop treatments tailored to the unique vulnerabilities of these populations.

Beyond issues of stratification, the overall quality of the evidence base remains limited. Many trials are small, underpowered, and of short duration, often relying on global depression scales that may lack sensitivity to mechanistically relevant symptom domains. Follow-up periods are frequently insufficient, and few studies incorporate disease specific biomarkers. These methodological constraints likely contribute to inconsistent findings and may underestimate the therapeutic potential of biologically informed interventions.

In interpreting this review, it is important to acknowledge several limitations. As a narrative synthesis, this review is subject to selection bias and does not include a formal risk-of-bias assessment, limiting the ability to systematically evaluate study quality. Second, for many therapeutic classes, evidence is largely derived from non-neurodegenerative populations, and the extent to which these findings generalise to AD and PD remains uncertain. Third, clinical studies in neurodegenerative populations are often characterised by small sample sizes, short follow-up, and limited replication, constraining confidence in durability and real-world applicability. Fourth, much of the mechanistic evidence is based on cross-sectional, case–control, or preclinical data rather than biomarker-embedded interventional studies. Finally, depression in AD and PD is phenotypically heterogeneous, and differences in symptom profile, disease stage, and comorbidity complicate comparisons across studies and limit the interpretability of aggregate outcomes. These limitations highlight the need for larger, biomarker-informed, and longitudinally designed trials in neurodegenerative populations. Nevertheless, the convergence of molecular, circuit, and behavioural findings provides a coherent framework to guide future investigation.

Depression in neurodegenerative disease is an area that is well placed to answer intersect evolving debates in psychiatry including the role of neuroinflammation, network psychiatry, and transdiagnostic psychopharmacology. Across modalities, several mechanistic themes emerge as particularly promising: glutamatergic modulation and synaptic plasticity enhancement; restoration of reward circuitry through dopaminergic and KCNQ channel modulation; and receptor-specific serotonergic targeting (e.g., 5-HT2A and 5-HT4 pathways). Therapeutically, extended-release ketamine formulations, circuit-informed neuromodulatory approaches, and precision receptor-targeted strategies represent feasible and testable avenues for disease-specific intervention. However, progress will likely depend on embedding biological stratification within trial design and integration of behavioural and neuroimaging markers within interventional studies, with further understanding of the disease specific dynamic evolution of psychiatric symptoms. Future trials of disease-modifying therapies must include depressive outcomes, and we should embrace the complexity of depression by incorporating dimensional measures as hypothesis driven primary outcomes, not just total depressive symptoms scores. This strategy may not only improve outcomes for patients with AD and PD, but also refine our broader understanding of depression as a disorder of distributed neural systems.

## Conclusion

There is an urgent need for novel and effective treatments for depression in neurodegenerative diseases, an area that offers real opportunity to significantly improve quality of life and prognosis for this patient group. While recent decades have seen substantial advances in the mechanistic understanding of depression and expanded the therapeutic landscape, patients with depression in the context of neurodegeneration have remained overlooked.

In AD and PD, depressive syndromes emerge within the context of defined neuropathology and progressive neurocognitive network disruption. Persisting with treatment paradigms developed in younger populations—without accounting for these disease-specific mechanisms—risks perpetuating therapeutic stagnation. Yet few areas of psychiatry offer such a clear opportunity for mechanism-based innovation. By bridging insights across pharmacological, neural, cognitive, and behavioural levels, depression in neurodegenerative disease could serve as a model for shifting psychiatry from symptom-based prescribing toward targeted, biologically informed treatment. Progress would not only address a major unmet clinical need, but also help define a more precise and mechanistically grounded future for the field.

## Supplementary information

Supplement

## Data Availability

Data are available on request.
