# Supplementary material for: Depression in neurodegenerative disease: neurobiological mechanisms and emerging treatments
Source: Mol Psychiatry. 2026 Jul 6;31(11):6603–21. doi: 10.1038/s41380-026-03728-8 (PMC13668706; doi:10.1038/s41380-026-03728-8)
Supplement: Supplementary file 1 [file 41380_2026_3728_MOESM1_ESM.docx]

**Supplement**

**Figure 1 method:**

To summarise differences in receptor availability between individuals with Alzheimer’s disease (AD) and healthy controls (HC), we extracted standardised mean differences (SMDs) in binding potential (BPND) from positron emission tomography (PET) studies for each receptor and brain region of interest (see Table S1 for included studies and effect sizes).

Studies contributing to Figure 1 were identified through existing systematic reviews and meta-analyses of PET imaging in AD. From these sources, we extracted study-level data reporting receptor- or transporter-specific binding potential (BPND or equivalent measures) in AD and HC groups. Where necessary, original articles were consulted to verify sample sizes, regional definitions, and numerical values.

If multiple publications reported overlapping cohorts, the most comprehensive or methodologically detailed dataset was retained to avoid duplication. Only studies reporting sufficient data to calculate SMDs were included. This approach was intended to provide a quantitative synthesis of previously published PET findings to facilitate cross-target comparison, rather than to constitute a formal systematic review of the PET literature.

Where multiple independent studies reported data for the same receptor within the same brain region, we conducted a meta-analysis using a random-effects model implemented in the *metafor* package in R. Study-level effect sizes (Cohen’s d) and corresponding variances were entered as inputs. Where raw group means and standard deviations were available, SMDs and variances were calculated using standard formulas. Where only a single study contributed data for a given receptor–region combination, the individual study estimate was retained. The resulting pooled or single-study SMDs were used to generate the figure summarising binding differences across targets and regions, as indicated in Table S1.

**Table S1: Summary of PET Binding Differences in Alzheimer's Disease vs. Healthy Controls**

Meta-analytic estimates are indicated in the 'Meta-analysed' column. SE = Standard Error.

| **Brain Region** | **Receptor** | **Study** | **N (AD)** | **N (HC)** | **SMD** | **Variance** | **Meta-analysed** | **Pooled SMD** | **Pooled variance** |
| --- | --- | --- | --- | --- | --- | --- | --- | --- | --- |
| Prefrontal cortex | 5-HT2A | Meltzer et al. 1999 | 9 | 10 | 0.6 | 0.22 | ✔ | 0.77 | 0.27 |
| Prefrontal cortex | 5-HT2A | Marner et al. 2012 | 12 | 11 | 0.92 | 0.19 |  |  |  |
| Prefrontal cortex | 5-HT2A | Santhosh et al. 2009 | 9 | 8 | 0.75 | 0.25 |  |  |  |
| Prefrontal cortex | 5HTT | Marner et al. 2012 | 12 | 11 | 0.98 | 0.2 | - | - | - |
| Prefrontal cortex | α4β2 nAChR | Sabri et al. 2018 | 14 | 15 | 0.75 | 0.25 | - | - | - |
| Frontal cortex | 5-HT1A | Kepe et al. 2008 | 8 | 5 | 0.58 | 0.34 | - | - | - |
| Frontal cortex | 5-HT2A | Santhosh et al. 2009 | 9 | 8 | 0.59 | 0.25 | - | - | - |
| Frontal cortex | α4β2 nAChR | Tiepolt et al. 2021 | 9 | 11 | 0.5 | 0.21 | ✔ | 0.98 | 0.31 |
| Frontal cortex | α4β2 nAChR | Sabri et al. 2018 | 14 | 15 | 0.56 | 0.14 |  |  |  |
| Frontal cortex | α4β2 nAChR | Kendziorra et al. 2010 | 9 | 7 | 1.26 | 0.3 |  |  |  |
| Frontal cortex | α4β2 nAChR | Nakaizumi et al. 2018 | 20 | 10 | 1.74 | 0.2 |  |  |  |
| Frontal cortex | M4 | Wenping et al. 2022 | 10 | 12 | 1.03 | 0.21 | - | - | - |
| Frontal cortex | Acetylcholinesterase | Shinotoh et al. 2000 | 14 | 14 | 1.06 | 0.16 | - | - | - |
| Frontal cortex | mGluR5 | Treyer et al. 2020 | 9 | 10 | 0.04 | 0.21 | ✔ | 0.14 | 0.31 |
| Frontal cortex | mGluR5 | Ishibashi et al. 2019 | 10 | 12 | 0.23 | 0.18 |  |  |  |
| Frontal cortex | mGluR2 | Ishibashi et al. 2019 | 10 | 12 | 0.45 | 0.19 | - | - | - |
| Parietal cortex | 5-HT1A | Kepe et al. 2009 | 8 | 5 | 1.09 | 0.37 | - | - | - |
| Parietal cortex | 5-HT2A | Meltzer et al. 1999 | 9 | 10 | 0.43 | 0.22 | ✔ | 0.58 | 0.27 |
| Parietal cortex | 5-HT2A | Marner et al. 2012 | 12 | 11 | 0.91 | 0.19 |  |  |  |
| Parietal cortex | 5-HT2A | Santhosh et al. 2009 | 9 | 8 | 0.34 | 0.24 |  |  |  |
| Parietal cortex | 5HTT | Marner et al. 2012 | 12 | 11 | -0.1 | 0.17 | - | - | - |
| Parietal cortex | α4β2 nAChR | Tiepolt et al. 2021 | 9 | 11 | 0.61 | 0.21 | ✔ | 0.55 | 0.25 |
| Parietal cortex | α4β2 nAChR | Sabri et al. 2018 | 14 | 15 | 0.4 | 0.14 |  |  |  |
| Parietal cortex | α4β2 nAChR | Kendziorra et al. 2010 | 9 | 7 | 0.74 | 0.27 |  |  |  |
| Parietal cortex | Acetylcholinesterase | Shinotoh et al. 2000 | 14 | 14 | 1.61 | 0.19 | - | - | - |
| Parietal cortex | mGluR5 | Treyer et al. 2020 | 9 | 10 | 0.08 | 0.21 | - | - | - |
| Occipital cortex | 5-HT2A | Meltzer et al. 1999 | 9 | 10 | 0.37 | 0.21 | ✔ | 0.73 | 0.36 |
| Occipital cortex | 5-HT2A | Marner et al. 2012 | 12 | 11 | 1.44 | 0.22 |  |  |  |
| Occipital cortex | 5-HT2A | Santhosh et al. 2009 | 9 | 8 | 0.37 | 0.24 |  |  |  |
| Occipital cortex | 5HTT | Marner et al. 2012 | 12 | 11 | 1.5 | 0.22 | - | - | - |
| Occipital cortex | α4β2 nAChR | Sabri et al. 2018 | 14 | 15 | -0.03 | 0.14 | ✔ | 0.2 | 0.31 |
| Occipital cortex | α4β2 nAChR | Kendziorra et al. 2010 | 9 | 7 | 0.63 | 0.27 |  |  |  |
| Occipital cortex | Acetylcholinesterase | Shinotoh et al. 2000 | 14 | 14 | 1.31 | 0.17 | - | - | - |
| Occipital cortex | mGluR5 | Treyer et al. 2020 | 9 | 10 | -0.34 | 0.21 | - | - | - |
| Occipital cortex | mGluR3 | Ishibashi et al. 2019 | 10 | 12 | -0.02 | 0.18 | - | - | - |
| Temporal lobe | 5-HT1A | Kepe et al. 2010 | 8 | 5 | 0.55 | 0.34 | - | - | - |
| Temporal lobe | 5-HT2A | Meltzer et al. 1999 | 9 | 10 | 0.92 | 0.23 | ✔ | 1.01 | 0.28 |
| Temporal lobe | 5-HT2A | Marner et al. 2012 | 12 | 11 | 1.17 | 0.2 |  |  |  |
| Temporal lobe | 5-HT2A | Santhosh et al. 2009 | 9 | 8 | 0.92 | 0.26 |  |  |  |
| Temporal lobe | 5HTT | Marner et al. 2012 | 12 | 11 | 0.14 | 0.17 | - | - | - |
| Temporal lobe | α4β2 nAChR | Tiepolt et al. 2021 | 9 | 11 | 0.78 | 0.22 | ✔ | 0.78 | 0.27 |
| Temporal lobe | α4β2 nAChR | Sabri et al. 2018 | 14 | 15 | 0.45 | 0.14 |  |  |  |
| Temporal lobe | α4β2 nAChR | Kendziorra et al. 2010 | 9 | 7 | 1.49 | 0.32 |  |  |  |
| Temporal lobe | M4 | Wenping et al. 2022 | 10 | 12 | 2.25 | 0.3 | - | - | - |
| Temporal lobe | Acetylcholinesterase | Shinotoh et al. 2000 | 14 | 14 | 1.7 | 0.19 | - | - | - |
| Temporal lobe | mGluR5 | Treyer et al. 2020 | 9 | 10 | 0.04 | 0.21 | - | - | - |
| Temporal lobe | mGluR7 | Ishibashi et al. 2019 | 10 | 12 | -0.5 | 0.19 | - | - | - |
| Striatum | 5-HT2A | Meltzer et al. 1999 | 9 | 10 | 1.93 | 0.31 | ✔ | 0.75 | 0.55 |
| Striatum | 5-HT2A | Marner et al. 2012 | 12 | 11 | 0.25 | 0.18 |  |  |  |
| Striatum | 5-HT2A | Santhosh et al. 2009 | 9 | 8 | 0.18 | 0.24 |  |  |  |
| Striatum | 5HTT | Marner et al. 2012 | 12 | 11 | 0.4 | 0.18 | - | - | - |
| Striatum | D1 | Kemppainen et al. 2000 | 10 | 8 | 1.63 | 0.3 | - | - | - |
| Striatum | D2 | Kemppainen et al. 2000 | 10 | 8 | 0.38 | 0.23 | - | - | - |
| Striatum | α4β2 nAChR | Sabri et al. 2018 | 14 | 15 | 0.24 | 0.14 | ✔ | 0.69 | 0.52 |
| Striatum | α4β2 nAChR | Kendziorra et al. 2010 | 9 | 7 | 1.29 | 0.31 |  |  |  |
| Striatum | M4 | Wenping et al. 2022 | 10 | 12 | -0.05 | 0.18 | - | - | - |
| Striatum | mGluR5 | Treyer et al. 2020 | 9 | 10 | 0.3 | 0.21 | - | - | - |
| Striatum | VMAT-2 | Siderowf et al. 2014 | 10 | 5 | -0.35 | 0.3 | - | - | - |
| Raphe nuclei | 5-HT1A | Kepe et al. 2007 | 8 | 5 | 1.54 | 0.42 | - | - | - |
| Raphe nuclei | 5HTT | Marner et al. 2012 | 12 | 11 | 0.17 | 0.17 | - | - | - |
| Anterior cingulate cortex | 5-HT2A | Meltzer et al. 1999 | 9 | 10 | 1.45 | 0.27 | ✔ | 1.21 | 0.28 |
| Anterior cingulate cortex | 5-HT2A | Marner et al. 2012 | 12 | 11 | 1.12 | 0.2 |  |  |  |
| Anterior cingulate cortex | 5-HT2A | Santhosh et al. 2009 | 9 | 8 | 1.1 | 0.27 |  |  |  |
| Anterior cingulate cortex | 5HTT | Marner et al. 2012 | 12 | 11 | 0.6 | 0.18 | - | - | - |
| Anterior cingulate cortex | α4β2 nAChR | Tiepolt et al. 2021 | 9 | 11 | 0.54 | 0.21 | ✔ | 0.59 | 0.26 |
| Anterior cingulate cortex | α4β2 nAChR | Sabri et al. 2018 | 14 | 15 | 0.36 | 0.14 |  |  |  |
| Anterior cingulate cortex | α4β2 nAChR | Kendziorra et al. 2010 | 9 | 7 | 1.14 | 0.29 |  |  |  |
| Anterior cingulate cortex | mGluR5 | Treyer et al. 2020 | 9 | 10 | 0.15 | 0.21 | - | - | - |
| Posterior cingulate cortex | 5-HT1A | Kepe et al. 2011 | 8 | 5 | 0.73 | 0.35 | - | - | - |
| Posterior cingulate cortex | α4β2 nAChR | Tiepolt et al. 2021 | 9 | 11 | 0.31 | 0.2 | ✔ | 0.64 | 0.41 |
| Posterior cingulate cortex | α4β2 nAChR | Sabri et al. 2018 | 14 | 15 | 0.24 | 0.14 |  |  |  |
| Posterior cingulate cortex | α4β2 nAChR | Kendziorra et al. 2010 | 9 | 7 | 1.65 | 0.34 |  |  |  |
| Posterior cingulate cortex | Acetylcholinesterase | Shinotoh et al. 2000 | 14 | 14 | 1.06 | 0.16 | - | - | - |
| Posterior cingulate cortex | mGluR5 | Treyer et al. 2020 | 9 | 10 | -0.05 | 0.21 | - | - | - |
| Insula | 5-HT2A | Marner et al. 2012 | 12 | 11 | 1.24 | 0.21 | - | - | - |
| Insula | 5HTT | Marner et al. 2012 | 12 | 11 | 0.13 | 0.17 | - | - | - |
| Insula | mGluR5 | Treyer et al. 2020 | 9 | 10 | -0.39 | 0.22 | - | - | - |
| Thalamus | 5-HT2A | Santhosh et al. 2009 | 9 | 8 | 0.1 | 0.24 | - | - | - |
| Thalamus | α4β2 nAChR | Sabri et al. 2018 | 14 | 15 | 0.72 | 0.15 | ✔ | 0.95 | 0.34 |
| Thalamus | α4β2 nAChR | Kendziorra et al. 2010 | 9 | 7 | 0.46 | 0.26 |  |  |  |
| Thalamus | α4β2 nAChR | Nakaizumi et al. 2018 | 20 | 10 | 1.62 | 0.19 |  |  |  |
| Thalamus | Acetylcholinesterase | Shinotoh et al. 2000 | 14 | 14 | 0.81 | 0.15 | - | - | - |
| Thalamus | mGluR5 | Treyer et al. 2020 | 9 | 10 | 0.0 | 0.21 | - | - | - |
| Amygdala | α4β2 nAChR | Sabri et al. 2018 | 14 | 15 | 0.46 | 0.14 | - | - | - |
| Amygdala | Acetylcholinesterase | Shinotoh et al. 2000 | 14 | 14 | 1.53 | 0.18 | - | - | - |
| Amygdala | mGluR5 | Treyer et al. 2020 | 9 | 10 | 1.46 | 0.27 | - | - | - |
